# Anti-histone and anti-nucleosome rather than anti-dsDNA antibodies associate with IFN-induced biomarkers in Sudanese and Swedish SLE patients

**DOI:** 10.1093/rheumatology/keae134

**Published:** 2024-03-09

**Authors:** Sahwa Elbagir, NasrEldeen A Mohammed, Vilija Oke, Anders Larsson, Jan Nilsson, Amir Elshafie, Elnour M Elagib, Musa A M Nur, Iva Gunnarsson, Elisabet Svenungsson, Johan Rönnelid

**Affiliations:** Department of Immunology, Genetics and Pathology, Uppsala University, Uppsala, Sweden; Faculty of Medical Laboratory Sciences, Al Neelain University, Khartoum, Sudan; Division of Rheumatology, Department of Medicine Solna, Karolinska Institute, Karolinska University Hospital, Stockholm, Sweden; Centre for Rheumatology, Academic Specialist Centre, Stockholm, Sweden; Department of Medical Sciences, Section of Clinical Chemistry, Uppsala University, Uppsala, Sweden; Department of Experimental Medical Science, Lund University, Lund, Sweden; Department of Immunology, Genetics and Pathology, Uppsala University, Uppsala, Sweden; Department of Clinical Immunology and Transfusion Medicine, Linköping University Hospital, Linköping, Sweden; Rheumatology Unit, Military Hospital, Omdurman, Sudan; Rheumatology Unit, Alribat University Hospital, Khartoum, Sudan; Division of Rheumatology, Department of Medicine Solna, Karolinska Institute, Karolinska University Hospital, Stockholm, Sweden; Division of Rheumatology, Department of Medicine Solna, Karolinska Institute, Karolinska University Hospital, Stockholm, Sweden; Department of Immunology, Genetics and Pathology, Uppsala University, Uppsala, Sweden

**Keywords:** anti-chromatin antibodies, anti-dsDNA, SLE, interferon, proteome analysis, Africa

## Abstract

**Objectives:**

In SLE, anti-dsDNA can co-occur with autoantibodies against other chromatin components, like histones and nucleosomes. These antibodies induce type-1 interferon production, a hallmark of SLE. We measured ANA sub-specificities and investigated their associations to inflammatory biomarkers including interferon-regulated chemokines.

**Methods:**

We included 93 Sudanese and 480 Swedish SLE patients and matched controls (*N* = 104 + 192). Autoantibodies targeting ANA sub-specificities: dsDNA, Sm, Sm/U1RNPcomplex, U1RNP, SSA/Ro52, SSA/Ro60, SSB/La, ribosomal P, PCNA and histones were quantified in all subjects, anti-nucleosome only in the Swedish patients, with a bead-based multiplex immunoassay. Levels of 72 plasma biomarkers were determined with the Proximity Extension Assay technique or ELISA.

**Results:**

Among Sudanese patients, the investigated antibodies were significantly associated with 9/72 biomarkers. Anti-histone antibodies showed the strongest positive correlations with MCP-3 and S100A12 as well as with interferon I-inducible factors MCP-1 and CXCL10. Anti-dsDNA antibodies were associated with CXCL10 and S100A12, but in multivariate analyses, unlike anti-histone, associations lost significance.

Among Swedish patients, MCP-1, CXCL10, and SA100A12 also demonstrated stronger associations to anti-histone and anti-nucleosome antibodies, compared with anti-dsDNA and other ANA sub-specificities. In multiple regression models, anti-histone/nucleosome retained the strongest associations. When excluding anti-histone or anti-nucleosome positive patients, the associations between MCP-1/CXCL10 and anti-dsDNA were lost. In contrast, when excluding anti-dsDNA positive patients, associations with anti-histone and anti-nucleosome remained significant.

**Conclusion:**

In two cohorts of different ethnical origins, autoantibodies targeting chromatin correlate stronger with IFN-induced inflammatory biomarkers than anti-dsDNA or other ANA sub-specificities. Our results suggest that anti-histone/nucleosome autoantibodies may be the main drivers of type-1 interferon activity in SLE.

Rheumatology key messages

- Antibodies against chromatin components associate stronger with interferon I-induced chemokines than anti-dsDNA antibodies.
- Similar findings seen in Sudanese and Swedish patients indicate a common association to SLE pathogenesis.

## Introduction

Systemic lupus erythematosus (SLE) is an autoimmune disease characterized by autoantibody production and impaired clearance of unviable cell remnants and immune complexes (IC). Autoantibodies targeting chromatin can either be directed against single structural components or the whole nucleosome [1]. Among all anti-nuclear antibodies (ANA), anti-dsDNA antibodies are considered as essential biomarkers in disease classification and assessment of disease activity [2]. Nevertheless, the primary antigenic stimulant to these autoantibodies can either be free DNA and/or chromatin-associated proteins like histones [3]. Histones in complexes with or without DNA enhance T and B cell responses in SLE, and can consequently augment production of anti-dsDNA or anti-chromatin autoantibodies [4, 5].

IC activate complement and induces the production of inflammatory mediators which are involved in biological networks that contribute to SLE pathophysiology. IC containing antibodies bound to RNA and DNA from apoptotic/unviable cells can bind Toll-like receptors (TLR) 7 and 9 in plasmacytoid dendritic cells and induce type I interferon (IFN) production [6–8]. Neutrophil extracellular traps (NETs) are net-like structures composed of DNA-histone complexes and proteins released by activated neutrophils [9]. NETs constituents can induce an IFN signature via TLR 9 binding [8]. Type I and II IFNs in turn promote the expression of a number of inflammatory mediators including MCP-1, CXCL10, CCL19, SIGLEC-1 and galectin-9 [10, 11]. IFN type I pathway activity can be estimated by measurement of IFN gene expression in peripheral blood cells, results are reported as IFN scores, which are based on the number of activated INF-induced genes [8, 12]. Commonly, type I IFN activity is measured by the expression of IFN-sensitive genes in reporter cells stimulated with patient serum [13, 14]. Quantification of circulating IFN-regulated chemokines can likewise depict IFN activity [15, 16]. IFN chemokine scores are more feasible and, in some studies, shown to perform better than IFN gene expression scores in predicting SLE disease activity [15].

To the best of our knowledge, there are no SLE proteome analysis studies from Africa elucidating associations between autoantibodies and disease phenotypes. We have previously reported an increased accumulation of anti-histone and anti-dsDNA autoantibodies in circulating IC from Sudanese SLE patients compared with corresponding levels in Swedish patients [17]. We also showed that Sudanese SLE patients are younger at diagnosis and experience more organ damage compared with Swedes [18]. In the current study, we performed a comprehensive proteome profiling including IFN-induced chemokines in plasma samples from Sudanese SLE patients and studied the correlation to the ANA profile, in an attempt to depict possible autoantibody patterns that might influence biomarker levels. To explore to what extent our findings were common to lupus in general, we performed parallel validation analyses in Swedish SLE patients.

## Methods

### Study cohorts

Ninety-three consecutive Sudanese and 480 Swedish SLE patients classified according to the 1982 revised (ACR) criteria [19, 20], were included. One hundred and four Sudanese healthy controls were matched for age and sex. Swedish population controls (*n* = 192) matched for age, sex and residential area were identified from population registries. More descriptive data of these cohorts are detailed in previous publications [18, 21].

All patients gave written informed consent to participate in the study. The Ethics Committees of Alribat University Hospital, Khartoum, Sudan and Omdurman Military Hospital, Omdurman, Sudan gave approval for the Sudanese cohort/study and the Karolinska University Hospital Ethics Committee approved the Swedish cohort/study.

### Autoantibody and proteomics assays

Quantification of ANA sub-specificities: dsDNA, Sm, the Sm/U1RNP complex, U1RNP, SSA/Ro52, SSA/Ro60, SSB/La, ribosomal P antigen, proliferating cell nuclear antigen (PCNA) and histones in serum was performed using an addressable laser bead immunoassay (ALBIA), (Fidis connective tissue profile, Theradiag, Marne la Vallee, France) according to the instructions of the manufacturer. As a dsDNA antigen, the Theradiag Fidis assay uses E. coli-based recombinant circular plasmid DNA whereas the Bioplex assay uses PCR-produced biotinylated DNA. The Theradiag assay uses a mix of purified H1, H3, H2B, H2A and H4 histones whereas the Bioplex assay uses calf thymus nuclear extract as chromatin/nucleosome antigen. The 98^th^ percentile values of the respective national controls were used as cutoffs for a positive reaction [18]. Another ALBIA (BioPlex 2200 system ANA screen, Bio-Rad, Hercules, CA, USA) was performed at the Karolinska University Hospital Laboratory for anti-dsDNA and anti-nucleosome antibody quantification in the Swedish cohort; cutoffs were determined using a specificity of 97.5% and 100% respectively.

Ninety-two inflammatory biomarkers were quantified in plasma with a proximity extension assay (PEA); using the targeted inflammation panel in the Sudanese cohort and the cardiovascular CVD I panel in the Swedish cohort (OLINK proteomics, Uppsala Sweden). Protein concentrations were reported as log2 transformation in a normalized protein expression (NPX) scale (https://olink.com). Proteins detected in >75% of the Sudanese samples (i.e. values equal or above the lowest detection limit) were included in the analyses (*n* = 72/92). A list of the studied proteins and their abbreviations is shown in Supplementary Table S1, available at *Rheumatology* online. Biomarkers with significant findings in the Sudanese cohort were evaluated in the Swedish cohort using the CVD I OLINK panel. In the Swedish cohort, CXCL10 was not part of the CVD panel, therefore levels were determined using ELISA in 311 of the Swedish patients. CXCL10 levels were analyzed as log values in parallel to the other inflammatory biomarkers.

### Statistical analysis

Autoantibodies were used as dichotomous variables and log-transformed biomarker levels as continuous variables. Differences in biomarker levels between two groups in the full SLE cohorts (Sudanese and Swedish, respectively) were studied using the Students’ *t-test*. The Benjamini-Hochberg False Discovery Rate (FDR) method for multiple testing correction was applied and a difference was considered significant if the adjusted *P*-value was <0.05. In the initial inflammatory biomarker screening these techniques were combined using the response screening platform in the JMP statistical software (SAS Institute, Cary, NC, USA). Multiple linear regression analyses including the variables showing significance in univariate analyses were adjusted for age and sex. Swedish patients were stratified for the occurrence of anti-dsDNA and anti-histone or anti-nucleosome antibodies. Biomarker concentrations were more skewed in these stratified subgroups compared with the full patient group; therefore, the Mann–Whitney test was used to determine differences after stratification. All statistical analyses were conducted using JMP.

## Results

### The sudanese cohort

**Figure 1.**
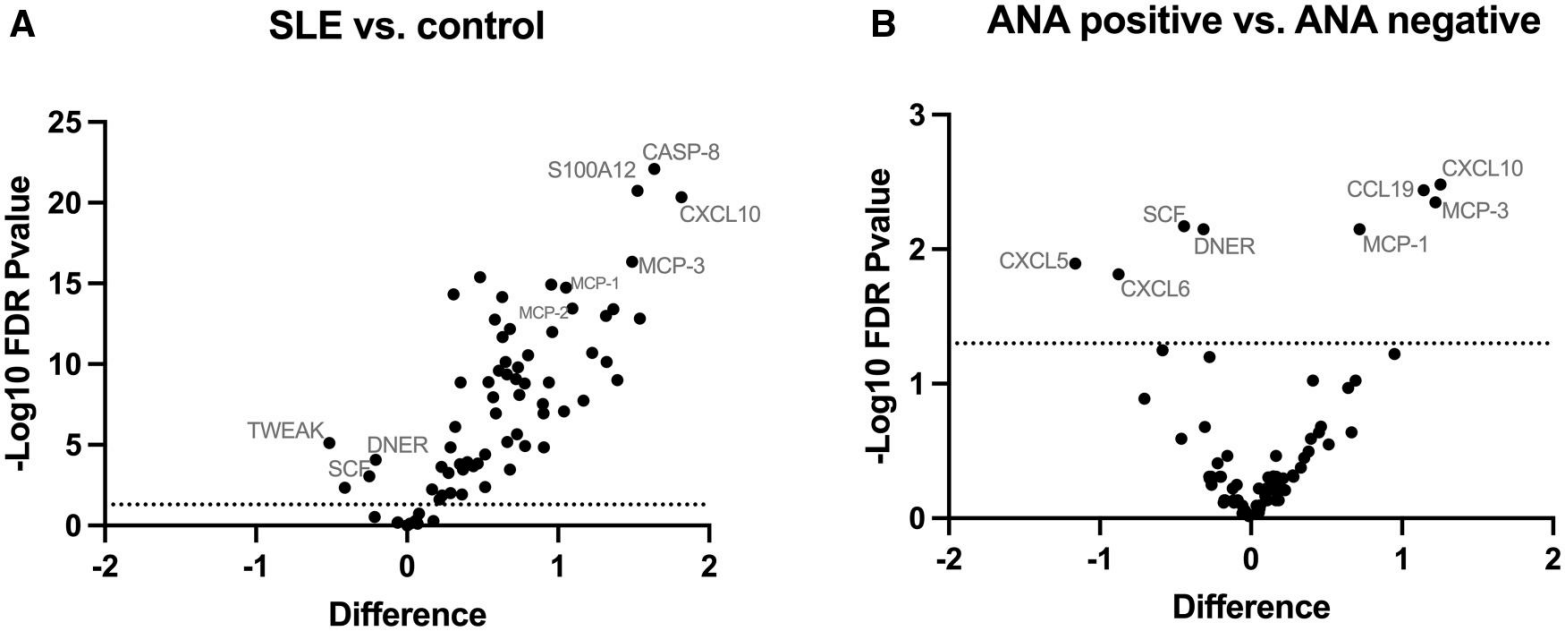
Volcano plot showing distribution of 72 inflammatory proteins in Sudanese cohort. Comparisons in SLE patients (*n* = 93) *vs* controls (*n* = 104), (A), and in patients positive for any ANA-associated autoantibody (*n* = 63) *vs* autoantibody negative patients (*n* = 30), (B). Significant protein level differences are demonstrated above the dotted line which represent FDR LogWorth with *P*-value <0.05. Benjamini-Hochberg method was used to correct for multiple testing. The X-axis represent the difference in means comparisons of studied proteins

Among SLE patients, nine individual proteins CXCL10, MCP-1, MCP-3, MCP-2, SLAMF1, CCL19, S100A12, DNER and SCF showed significant associations with at least one specific ANA-associated autoantibody, see Table 1. These inflammatory proteins were also differentially expressed in SLE patients compared with controls with high significant differences noted for CXCL10, MCP-1, MCP-3 and S100A12, Fig. 1A. Likewise, concentrations for CXCL10, MCP-1, MCP-3 and CCL19 were significantly increased in patients positive for any ANA-associated antibody *vs* antibody-negative patients, Fig. 1B. Full data for all individual 72 protein biomarkers are shown in Supplementary Table S2a–c, available at *Rheumatology* online.

**Table 1.** Association of ANA sub-specificities with levels of inflammatory proteins in Sudanese patients (*n* = 93)

	MCP-1		MCP-3		CXCL10		CCL19		MCP-2		S100A12		SLAMF-1		SCF		DNER	
Autoantibody Specificity (% of cohort positive)	mean (median) +/−	R^2^/FDRP	mean (median) +/−	R^2^/FDRP	mean (median) +/−	R^2^/FDRP	mean (median) +/−	R^2^/FDRP	mean (median) +/−	R^2^/FDRP	mean (median) +/−	R^2^/FDRP	mean (median) +/−	R^2^/FDRP	mean (median) +/−	R^2^/FDRP	mean (median) +/-	R^2^/FDRP
Anti-SSA/Ro52 (22%)	13.1(13.0)/12.6(12.5)	0.05/0.3	4.3(3.7)/3.4(3.0)	0.05/0.3	11.4(11.3)/10.9(10.7)	0.02/0.5	10.7(11.0)/9.9(9.9)	0.05/0.3	10.8(10.9)/10.6(10.5)	0.01/0.7	5.1(4.9)/5.0(4.8)	0.002/0.9	3.6(3.7)/3.7(3.7)	0.001/0.9	9.4(9.5)/9.8(9.9)	0.08/0.1	9.0(9.1)/9.3(9.3)	0.08/0.1
Anti-SSA/Ro60 (9.6%)	13.1(13.1)/12.7(12.6)	0.02/0.6	4.5(4.1)/3.6(3.1)	0.03/0.5	11.7(11.8)/10.9(10.7)	0.03/0.5	10.8(11.5)/10.0(9.9)	0.03/0.4	10.9(10.5)/10.6(10.6)	0.009/0.7	5.1(5.5)/5.0(4.8)	<0.001/0.9	3.7(3.8)/3.7(3.7)	<0.001/0.9	9.3(9.4)/9.8(9.9)	0.06/0.2	9.0(9.1)/9.3(9.3)	0.02/0.5
Anti-SSB (6.4%)	13.1(13.1)/12.7(12.6)	0.01/06	4.7(4.4)/3.6(3.1)	0.03/0.5	11.8(11.5)/11.0(10.7)	0.02/0.5	11.3(11.4)/10.0(9.9)	0.06/0.2	11.4(11.3)/10.6(10.5)	0.04/0.4	5.3(5.8)/5.0(4.8)	0.005/0.8	3.8(3.9)/3.7(3.7)	0.003/0.9	9.6(9.5)/9.7(9.9)	0.002/0.9	9.2(9.2)/9.2(9.3)	<0.001/0.9
Anti-Sm (34%)	13.0(12.9)/12.6(12.5)	0.04/0.4	4.3(4.1)/3.3(3.0)	0.09/0.09	11.7(11.7)/10.7(10.4)	0.12/0.04	10.6(10.8)/9.8(9.9)	0.08/0.1	11.0(10.8)/10.5(10.5)	0.05/0.3	5.3(5.5)/4.8(4.7)	0.04/0.4	3.7(3.7)/3.6(3.7)	0.009/0.7	9.5(9.6)/9.8(9.9)	0.06/0.2	9.1(9.0)/9.3(9.4)	0.09/0.1
Anti-SmRNP (32%)	13.1(13.0)/12.5(12.5)	0.09/0.09	4.1(3.5)/3.4(3.0)	0.04/0.3	11.6(11.5)/10.7(10.4)	0.09/0.1	10.7(10.9)/9.8(9.9)	0.09/0.1	11.0(10.9)/10.5(10.5)	0.07/0.1	5.1(5.0)/4.9(4.8)	0.005/0.8	3.8(3.7)/3.6(3.7)	0.01/0.7	9.6(9.8)/9.8(9.9)	0.02/0.6	9.1(9.1)/9.3(9.3)	0.08/0.1
Anti-U1RNP (25%)	13.2(12.8)/12.5(12.5)	0.09/0.09	4.4(3.7)/3.4(3.1)	0.09/0.09	11.8(11.7)/10.8(10.4)	0.09/0.08	10.8(11.2)/9.8(9.9)	0.1/0.07	11.2(11.0)/10.5(10.5)	0.11/0.07	5.4(5.5)/4.9(4.7)	0.04/0.4	3.8(3.8)/3.6(3.6)	0.04/0.4	9.4(9.6)/9.8(9.9)	0.09/0.1	9.0(9.0)/9.3(9.3)	0.12/0.04
**Anti-dsDNA (39%)**	13.0(12.9)/12.7(12.5)	0.04/0.4	4.3(3.8)/3.3(3.1)	0.1/0.08	11.7(11.4)/10.6(10.1)	0.13/0.03	10.4(10.6)/9.8(9.9)	0.04/0.3	10.9(10.9)/10.5(10.4)	0.04/0.3	5.6(5.6)/4.6(4.6)	0.13/0.04	3.8(3.7)/3.6(3.6)	0.02/0.6	9.5(9.7)/9.9(9.9)	0.1/0.07	9.2(9.2)/9.3(9.3)	0.02/0.5
**Anti-histone (11%)**	13.8(13.8)/12.6(12.5)	0.15/0.02	5.8(6.5)/3.4(3.1)	0.23/0.0003	12.7(13.1)/10.8(10.7)	0.16/0.01	11.2(11.3)/9.9(9.9)	0.08/0.1	11.5(11.2)/10.5(10.5)	0.08/0.1	6.3(6.5)/4.8(4.8)	0.14/0.02	4.2(4.2)/3.6(3.6)	0.14/0.02	8.9(8.8)/9.8(9.9)	0.23/0.0004	9.0(8.8)/9.3(9.3)	0.06/0.2
Anti-ribosomal P (18%)	13.4(13.1)/12.6(12.5)	0.12/0.04	4.7(3.5)/3.4(3.1)	0.1/0.08	11.8(11.3)/10.8(10.6)	0.06/0.2	11.1(11.3)/9.9(9.9)	0.12/0.04	11.4(11.1)/10.5(10.4)	0.14/0.03	5.3(4.8)/4.9(4.8)	0.01/0.7	3.9(4.0)/3.6(3.7)	0.06/0.2	9.4(9.7)/9.8(9.9)	0.07/0.1	9.1(9.1)/9.3(9.3)	0.03/0.5
Anti-PCNA (12%)	13.3(13.5)/12.6(12.5)	0.05/0.3	4.3(3.5)/3.6(3.1)	0.02/0.6	11.7(11.7)/10.9(10.7)	0.03/0.5	10.5(11.0)/10.0(9.9)	0.01/0.7	11.0(11.1)/10.6(10.5)	0.02/0.6	4.9(4.4)/5.0(4.8)	0.001/0.9	3.8(3.6)/3.7(3.7)	0.005/0.8	9.5(9.4)/9.7(9.9)	0.01/0.7	9.1(9.1)/9.3(9.3)	0.02/0.6

Relative concentrations are shown for the nine inflammatory proteins yielding different distribution among autoantibody positive *vs* autoantibody negative patients in initial response screening of all 72 investigated proteins. Protein concentrations are reported as Log2 values. Benjamini-Hochberg method was used to correct for multiple testing with significant adjusted *P*-value < 0.05 underlined.

Anti-histone antibodies, compared with other ANA specificities, showed the strongest biomarker associations; positively with IFN-induced biomarkers (CXCL10, MCP-1 and MCP-3; r^2^/FDRp: 0.16/0.01, 0.15/0.02 and 0.23/0.0003, respectively), S100A12 (0.14/0.02), SLAMF1 (0.14/0.02) and negatively with SCF (0.23/0.0004), see Table 1. Anti-dsDNA antibodies showed a less strong association with CXCL10 (0.13/0.03) and S100A12 (0.13/0.04). Positive associations were also seen between anti-ribosomal antibodies and MCP-1, MCP-2 and CCL19, and between anti-Sm and CXCL10. Anti-U1RNP is associated negatively with DNER. Parallel non-parametric response screening confirmed the central role of anti-histone antibodies, see Supplementary Tables S3a and b, available at *Rheumatology* online.

In multiple regression models that included age, sex and all ANA sub-specificities that had significant univariate biomarker associations, anti-histone remained as the antibody with the strongest association to biomarker concentrations, whereas associations to anti-dsDNA were lost (Table 2). Further adjustment for prednisolone use as an independent variable in the model did not affect antibody associations to proteins (data not shown).

**Table 2.** Multivariate analysis of association between autoantibodies and inflammatory proteins among Sudanese patients (*n* = 93)

	MCP-1	MCP-3	CXCL10	CCL19	MCP-2	S100A12	SLAMF1	SCF	DNER
	Std beta (p)	Std beta (p)	Std beta (p)	Std beta (p)	Std beta (p)	Std beta (p)	Std beta (p)	Std beta (p)	Std beta (p)
Anti-Sm			0.22(0.04)						
Anti-U1RNP									-0.35(0.0008)
Anti-dsDNA			0.17(0.1)			0.15(0.1)			
Anti-histone	0.34(0.0009)	0.45(<0.0001)	0.31(0.002)			0.27(0.007)	0.4(0.0001)	-0.46(<0.0001)	
Anti-ribosomal P	0.26(0.009)			0.33(0.001)	0.37(0.0003)				

ANA sub-specificities previously showing associations in univariate comparisons (Table 1) were included. Each analysis included all five autoantibodies as independent variables and one individual inflammatory protein as dependent variable, and were adjusted for age and sex. Significant associations are underlined.

### The Swedish cohort

Parallel quantification data for 4 proteins (MCP-1, CXCL10, SA100A12 and SCF) were available in the Swedish cohort and were used to validate the results obtained in the Sudanese cohort. In this larger cohort, many ANA specificities showed significant associations with all 4 proteins. Again, anti-histone positivity showed the strongest positive correlation to biomarkers, Table 3. These results were also confirmed with non-parametric testing, Supplementary Table S3c, available at *Rheumatology* online. In multiple regression analyses adjusted for age and sex and including the significant ANA-specificities reported in Table 3, anti-histone uniformly remained as the antibody with the strongest positive associations to levels of MCP-1, CXCL10 and S100A12, Table 4. Anti-Sm was also associated positively with MCP-1, anti-PCNA with S100A12 and anti-ribosomal p negatively with SCF, Table 4. As in the Sudanese cohort, further adjustment for prednisolone did not change these findings (data not shown).

**Table 3.** Association of ANA sub-specificities with inflammatory proteins among Swedish SLE patients (*n* = 480)

	MCP-1		CXCL10 (n = 311)		S100A12		SCF neg	
(antibody positive%)	mean (median) +/−	R^2^/FDRP	mean (median) +/−	R^2^/FDRP	mean (median) +/−	R^2^/FDRP	mean (median) +/−	R^2^/FDRP
Anti-SSA/Ro52 (27%)	3.2(3.0)/3.0(2.9)	0.01/0.03	5.6(5.5)/5.3(5.2)	0.03/0.002	1.3(0.9)/1.3(0.8)	<0.001/0.9	6.8(6.7)/6.9(7.0)	0.005/0.1
Anti-SSA/Ro60 (37%)	3.1(3.0)/2.9(2.9)	0.01/0.01	5.6(5.5)/5.2(5.1)	0.04/0.0008	1.5(0.9)/1.3(0.8)	0.004/0.2	6.9(6.8)/6.9(7.0)	0.01/0.02
Anti-SSB (18%)	3.1(3.0)/3.0(2.9)	0.002/0.3	5.5(5.4)/5.3(5.2)	0.01/0.08	1.2(0.6)/1.4(0.9)	0.003/0.2	6.8(6.8)/6.9(6.9)	0.002/0.4
Anti-Sm (17%)	3.3(3.1)/2.9(2.9)	0.03/0.0005	5.6(5.6)/5.3(5.2)	0.03/0.006	1.9(1.3)/1.2(0.8)	0.03/0.0004	6.6(6.6)/6.9(6.9)	0.03/0.0004
Anti-SmRNP (47%)	3.1(3.0)/2.9(2.9)	0.02/0.002	5.5(5.5)/5.2(5.1)	0.06/<0.0001	1.6(1.1)/1.1(0.6)	0.04/<0.0001	6.7(6.7)/7.0(7.0)	0.06/<0.0001
Anti-U1RNP (19%)	3.1(3.0)/3.0(2.9)	0.002/0.3	5.6(5.4)/5.3(5.2)	0.01/0.04	1.5(1.0)/1.3(0.8)	0.003/0.2	6.7(6.7)/6.9(7.0)	0.01/0.02
Anti-dsDNA (37%)	3.3(3.1)/2.9(2.8)	0.06/<0.0001	5.6(5.6)/5.2(5.2)	0.07/<0.0001	2.0(1.6)/1.0(0.6)	0.12/<0.0001	6.7(6.7)/6.9(7.0)	0.04/<0.0001
Anti-histone (31%)	3.4(3.3)/2.8(2.8)	0.09/<0.0001	5.7(5.7)/5.2(5.1)	0.08/<0.0001	2.2(1.8)/1.0(0.6)	0.15/<0.0001	6.7(6.7)/6.9(7.0)	0.02/0.001
Anti-ribosomal P (20%)	3.1(3.1)/2.9(2.9)	0.01/0.01	5.6(5.5)/5.3(5.2)	0.03/0.004	1.5(1.2)/1.3(0.8)	0.004/0.2	6.7(6.6)/6.9(7.0)	0.03/0.0005
Anti-PCNA (5%)	3.4(3.3)/3.0(2.9)	0.01/0.02	5.8(5.8)/5.3(5.2)	0.02/0.02	2.5(2.1)/1.3(0.8)	0.04/<0.0001	6.7(6.9)/6.9(6.9)	0.003/0.3

Log transformed concentrations are shown for four proteins that showed associations in the Sudanese SLE cohort (Table 2). T-test with Benjamini-Hochberg correction for multiple testing was used; significant adjusted *P* values <0.05 are underlined.

**Table 4.** Multivariate analysis of inflammatory proteins with ANA sub-specificities among Swedish patients (*n* = 480)

	MCP-1	CXCL10	S100A12	SCF
	Std beta (p)	Std beta (p)	Std beta (p)	Std beta (p)
Anti-SSA/Ro52	0.03(0.6)	0.09(0.2)		
Anti-SSA/Ro60	0.06(0.3)	0.11(0.1)		−0.08(0.06)
Anti-SSB				
Anti-Sm	0.11(0.02)	0.05(0.4)	0.07(0.2)	−0.09(0.1)
Anti-U1RNP		0.06(0.3)		−0.04(0.4)
Anti-dsDNA	0.11(0.05)	0.15(0.02)	0.16(0.003)	−0.11(0.06)
Anti-histone	0.23(<0.0001)	0.18(0.004)	0.27(<0.0001)	−0.04(0.4)
Anti-ribosomal P	0.01(0.8)	0.08(0.1)		−0.1(0.03)
Anti-PCNA	0.06(0.1)	0.08(0.1)	0.13(0.002)	

Analysis performed for variables that had previously significant univariate comparisons (Table 3). Number of patients with available CXCL10 data were 311. Anti-SmRNP antibodies were not included in the multiple regressions because of overlap positivity with anti-Sm/anti-U1RNP antibodies. Standardized β and P values were calculated using linear regression models adjusted for age and sex. Significant associations (*P* < 0.05) are underlined.

Using an independent autoantibody measurement assay, anti-nucleosome antibodies showed stronger associations with IFN-induced proteins (MCP-1 and CXCL10) than anti-dsDNA positivity, Table 5. This was also confirmed by multiple regression analyses adjusted for age and sex, Supplementary Table S4, available at *Rheumatology* online.

**Table 5.** Associations of inflammatory proteins with anti-dsDNA, anti-histone and anti-nucleosome antibodies

		MCP-1		CXCL10		S100A12		SCF	
*FIDIS*		mean (median) +/−	*P* value	mean (median) +/−	*P* value	mean (median) +/−	*P* value	mean (median) +/−	*P* value
**ALL patients *n* = 480**	**Anti-dsDNA**	**3.3(3.1)/2.9(2.8)**	**<0.0001**	**5.6(5.6)/5.2(5.2)**	**<0.0001**	**2.0(1.6)/1.0(0.6)**	**<0.0001**	**6.7(6.7)/6.9(7)**	**<0.0001**
**ALL patients *n* = 480**	**Anti-histone**	**3.4(3.3)/2.8(2.8)**	**<0.0001**	**5.7(5.7)/5.2(5.1)**	**<0.0001**	**2.2(1.8)/1.0(0.6)**	**<0.0001**	**6.7(6.7)/6.9(7)**	**0.0008**
**Anti-dsDNA positive patients excluded *n* = 180**	**Anti-histone +ve *n* = 29 (300)**	**3.1(3.0)/2.8(2.8**	**0.02**	**5.5(5.5)/5.1(5.1)**	**0.03**	**1.5(1.1)/0.9(0.6)**	**0.005**	**6.9(7)/6.9(6.9)**	**0.7**
**Anti-histone positive patients excluded *n* = 148**	**Anti-DsDNA +ve *n* = 61 (332)**	**2.9(2.8)/2.8(2.8)**	**0.7**	**5.4(5.2)/5.1(5.1)**	**0.1**	**1.3(0.9)/0.9(0.6)**	**0.009**	**6.8(6.9)/6.9(7)**	**0.04**
***Bioplex***									
**ALL patients *n* = 480**	**Anti-dsDNA**	**3.2(3.1)/2.9(2.8)**	**0.0003**	**5.6(5.5)/5.2(5.2)**	**0.005**	**1.9(1.5)/1.0(0.6)**	**<0.0001**	**6.8(6.8)/6.9(6.9)**	**0.03**
**ALL patients *n* = 480**	**Anti-Nucleosome**	**3.2(3.1)/2.8(2.8)**	**<0.0001**	**5.6(5.5)/5.2(5.1)**	**<0.0001**	**1.9(1.5)/0.9(0.6)**	**<0.0001**	**6.7(6.8)/6.9(7)**	**0.0003**
**Anti-dsDNA positive patients excluded *n* = 185**	**Anti-Nucleosome +ve *n* = 80 (295)**	**3.0(3.0)/2.8(2.8)**	**0.04**	**5.5(5.5)/5.1(5.1)**	**0.003**	**1.2(0.8)/0.9(0.6)**	**0.055**	**6.9(6.9)/6.9(7)**	**0.2**
**Anti-nucleosome positive patients excluded *n* = 215**	**Anti-DsDNA +ve *n* = 50 (265)**	**2.8(2.8)/2.8(2.8)**	**0.8**	**5.2(5.2)/5.1(5.1)**	**0.7**	**0.7(0.4)/0.9(0.6)**	**0.2**	**7(7.1)/6.9(7)**	**0.3**

Comparisons were performed among Swedish patients after stratification to separate autoantibody positive patient groups. Autoantibody data were available from two addressable laser bead assays, FIDIS and Bio-Rad, presented separately in the upper and lower parts of the table respectively. Statistical significance was calculated using Mann–Whitneýs *U* test due to the skewed distribution of data after stratification. Comparisons are demonstrated for associations between the specific ANA autoantibody and inflammatory proteins after exclusion of the respective patient groups shown in the first column. Significant associatons are underlined.

Using stratification analyses, excluding anti-histone or anti-nucleosome positive patients, the associations between anti-dsDNA and the biomarkers MCP-1 and CXCL10 were lost. In contrast, when excluding anti-dsDNA positive patients, the corresponding biomarker associations with anti-histone and anti-nucleosome remained significant, Table 5. SA100A12 associations with anti-dsDNA antibodies remained significant after the exclusion of anti-histone positive patients, but were lost when excluding anti-nucleosome positive patients.

## Discussion

Inflammatory biomarkers, predominantly IFN-regulated, associate stronger with anti-histone and anti-nucleosome autoantibodies than with anti-dsDNA and other ANA sub-specificities. Parallel results were obtained in Sudanese and Swedish SLE patients, and findings were confirmed with multivariate and stratification analyses. The anti-dsDNA-independent associations between anti-histone and anti-nucleosome with IFN-regulated chemokines, might imply that histones and/or chromatin as a whole play a more significant role than uncomplexed dsDNA as autoantigens driving IFN activity.

Previous studies have reported that type 1 IFN activity **is** measured directly as gene transcripts [22–24] or indirectly by reporter cell assays [25] associate with anti-Sm, anti-ribonucleoprotein (anti-RNP), anti-dsDNA and anti-SSA/SSB antibodies. A very recent study by Hubbard *et al.* demonstrated that IFN signatures correlate with anti-RNP and not anti-dsDNA antibodies in African American (AA) patients, but with both antibodies in patients of Caucasian ancestry [26]. It is noteworthy that most of these studies did not include anti-nucleosome or anti-histone antibodies in their analyses. Very few studies analysed antibodies against chromatin components (anti-dsDNA, anti-histone, anti-nucleosome), separately, to investigate correlations with inflammatory biomarkers in SLE. In a recent report by Enocsson *et al.* investigating surrogate biomarkers to type I IFN in SLE, the authors indicated that both CXCL10 and CCL19 correlate stronger with anti-histone antibodies than did antibodies against dsDNA, SmRNP and SSA (Fig. 3 in [11]). Furthermore, Oke *et al.* and Fayed *et al.* reported a lack of association between serum IFNα levels and anti-dsDNA antibodies in Swedish and Egyptian SLE patients, respectively [25, 27]. In another study where AA constituted 55% of the investigated SLE cohort, IFNα activity showed a stronger association with anti-chromatin antibodies than with anti-dsDNA antibodies and other ANA**-**specificities [28]. Anti-nucleosome antibodies and nucleosome-specific CD4 positive T cells appear earliest preceding anti-dsDNA in lupus nephritis [3, 29, 30]. Histone-specific T cells were previously shown to augment the production of anti-dsDNA in an SLE mice model [4]. In line with these findings, we identified 4 distinct SLE clusters based on autoantibody profiles. Anti- nucleosome/Sm/RNP/dsDNA antibodies dominated one of these clusters comprising 29% of the investigated patients. It was characterized by a distinct biomarker profile (including higher IFNγ levels), earlier disease onset, and more nephritis and was the only cluster associated to HLA-DRB1*15, indicating that we may be dealing with a distinct SLE subset [31]. With the cross-sectional design of our current study, we cannot determine whether autoantibodies induce inflammatory biomarkers, or vice versa. Nevertheless, in agreement with the large literature on IFN induction by autoantibody-containing IC; we hypothesize that anti-histone and anti-nucleosome antibodies act as IFN inducers [8, 32, 33].

IFN-associated biomarkers (CXCL10, MCP-1, MCP-3 and CCL19) were significantly increased in Sudanese patients positive for any ANA-associated specificity. Levels of these proteins also discriminate SLE patients from controls; with convincing statistical difference for CXCL10 and MCP-3. It has previously been demonstrated that SLE patients of African ancestry have a more distinct IFN signature as compared with Caucasian patients [15, 26], which might partly explain that SLE is more severe in these populations. MCP-3 more than MCP-1 and 2, and IFNγ-induced chemokines more than IFNγ levels characterized pre-flare compared with non-flare states among SLE patients in an AA cohort [34]. SLE classification was best predicted by IFNγ, MCP-3 and anti-chromatin antibodies in a previously studied pre-SLE cohort dominated by AA patients [28]. These findings agree with our current results where the most significant antibody associations as well as differences between patients and controls exist for MCP-3 and CXCL10.

We also find signals for the S100 protein S100A12 both in the Sudanese and Swedish cohorts. S100A12 is produced by neutrophil granulocytes and perhaps also by monocytes [35, 36]. S100A12 gene expression can be transiently induced by TNFα in monocytes and by IL-6 in the THP-1 macrophage cell line [37, 38]. A recent single-cell transcriptomics paper has found associations between S100A12 expression in classical monocytes and an interferon I signature in children with covid-19 encephalopathy [39], but S100A12 is not at presently considered to be part of an interferon signature.

SCF contributes mainly to hemopoiesis [40] and has been shown to be associated with markers of renal involvement in lupus [41]. In the current study, we found negative associations between SCF as well as DNER (Delta/NOTCH-like epidermal growth factor) with several autoantibodies including anti-U1RNP, anti-dsDNA and anti-histones. These proteins were also significantly lower in SLE patients compared with controls. In agreement with our results, Kitoh *et al.* showed that SCF associate negatively with levels of anti-RNP in SLE patients, but no significant difference in levels were found between patients and controls [42]. Down-regulation of SCF and DNER, when other inflammatory biomarkers are upregulated, has however been noted also in sepsis [43] and severe COVID-19 infection [44]. Inflammatory cytokines like IL-1α, IFNγ and TNFα can downregulate SCF expression [45, 46]. Therefore, we believe that the aberrant expression of SCF and DNER in our studies probably depends on common mechanisms regulating inflammation, and is not primarily associated with SLE.

Many more autoantibody specificities show significant associations in the Swedish lupus cohort as compared with the Sudanese group. We think this is primarily due to the large differences in cohort size between the Sudanese and Swedish cohorts as the same effect size will more easily be statistically significant when the number of individuals increases.

Our cross-sectional study design is a limitation. We could not determine whether anti-chromatin antibodies induce IFN-related chemokines, or vice versa. Also, patient stratification analyses were not possible in the Sudanese cohort due to the small subgroup sizes.

To our knowledge, this is the first study from the African continent that investigated inflammatory proteome profiles in SLE. Our results highlight the importance of anti-chromatin antibodies and we demonstrate that key inflammatory mediators correlate with specific SLE-related autoantibodies, and the results could be replicated in a larger Caucasian cohort. We believe these results will add more understanding to the complexity of the biological networks involved in SLE patients of different ethnicities. Furthermore, since autoantibody profiles, unlike chemokines, are available in the clinic, our results merit to be considered when selecting patients who may benefit from therapies blocking the type-I IFN receptor and downstream mediators [47].

## Supplementary Material

keae134_Supplementary_Data

## Data Availability

Data that support the findings of this study are not publicly available to protect the privacy of participants.
